# The Status of Laparoscopic Inguinal Hernia Surgery in Children: A Nationwide Assessment

**DOI:** 10.3390/children9030348

**Published:** 2022-03-03

**Authors:** Andrea Schmedding, Ahmad Alsweed, Oliver Muensterer, Johannes Leonhardt

**Affiliations:** 1 Department of Pediatric Surgery and Pediatric Urology, University Hospital Frankfurt, Goethe University Frankfurt, D-60590 Frankfurt, Germany; 2 Department of Pediatric Surgery and Pediatric Urology, Klinikum Braunschweig gGmbH, D-38126 Braunschweig, Germany; a.alsweed@klinikum-braunschweig.de (A.A.); j.leonhardt@klinikum-braunschweig.de (J.L.); 3 Department of Pediatric Surgery, Dr. von Hauner Children’s Hospital, LMU Klinikum, D-80337 Munich, Germany; oliver.muensterer@med.uni-muenchen.de

**Keywords:** inguinal hernia, child, laparoscopic inguinal hernia repair, pediatric surgery, minimal invasive

## Abstract

Inguinal hernia repair (IHR) is a common procedure in childhood. Laparoscopic IHR has been evolving for the last three decades. Although clear advantages have been shown, adaptation in Germany has been slow. We aim to study the current status of pediatric laparoscopic IHR. A survey was sent to all 89 pediatric surgical departments in Germany on current practices and preferences of open versus laparoscopic IHR. Two nationwide databases of administrative claims data from 2019 were analyzed and correlated with responses from the survey. A total of 56% of the pediatric surgical departments supplied data through the quality reports. The recall of our survey was 58% of all pediatric surgery departments. According to the pooled data, laparoscopic IHR was performed in 8.2% of all inpatients treated. Laparoscopic IHR was considered a training procedure in 48% of the departments. Five different laparoscopic techniques were described (most commonly percutaneous closure of the hernia under laparoscopic vision). The choice between open and laparoscopic IHR was mainly determined by the child’s age. Currently, only a minority of German children undergo inguinal hernia repair by laparoscopy. More training opportunities in the form of hands-on and video workshops may lead to more widespread employment of the laparoscopic technique.

## 1. Introduction

Inguinal hernia repair (IHR) is the most common surgical procedure in childhood. The incidence of inguinal hernia under the age of 18 years is estimated to be between 0.8 and 4.4% [1]. It is much more common in males (male to female ratio is 5 to 1) [2]. In males, the peak incidence is in the first year, whereas in girls it is at around 5 years of life [3].

Most of the inguinal hernias in children are indirect [2]. Pediatric inguinal hernia repair usually comprises dissection of the hernia sac at the inner inguinal ring followed by high ligation. Open IHR is an extra-abdominal procedure with a high success rate and few complications. It is still considered the most commonly performed approach in children.

Laparoscopic IHR has evolved since its introduction in the early 1990s [1]. Since then, different techniques have been described, including a transabdominal three-port technique with suturing the neck of the hernia sack [4] and single-port laparoscopic percutaneous extraperitoneal closure assisted by optical forceps [5].

Clear advantages for laparoscopic inguinal hernia repair have been demonstrated in terms of shorter operative times for bilateral hernias [6], a reduction in metachronous hernia development [7] and the opportunity to explore and proactively repair a contralateral hernia [8]. Total postoperative complications have been reported less in laparoscopic IHR [9], as well as a decreased risk of postoperative iatrogenic ascending testis. The main disadvantage is an increased risk of wound infection [8]. Moreover, a recent meta-analysis found no difference regarding overall mean operative time, postoperative pain and recurrence rate [10].

In an international survey from 2012 with respondents from 46 countries, the preferred approach for inguinal hernia was open in 83% and laparoscopic in 4% [11]. In a recent survey from Denmark, 14% of respondents considered laparoscopic IHR in children below two years of age, while 34% considered it in children older than twelve years of age [12].

Although many studies have been published comparing laparoscopic and open IHR, it is unclear how and how often laparoscopic technique is used in Germany on a national level. Our goal for this study was therefore to evaluate if laparoscopic inguinal hernia repair remained a niche technique performed by a select group of highly specialized pediatric surgeons or if it had evolved into an accepted mainstream therapy.

Germany is a country with decentralized pediatric surgical care [13], comprising of 89 departments, 42 small units and 100 private practices distributed throughout the country [14]. Currently there are more than 700 active pediatric surgeons in practice. Furthermore, IHR in children is performed by pediatric surgeons and surgeons of other specialties, such as urologists and general surgeons. Inguinal hernia repairs are either performed in hospitals or private practices. They are mainly performed by pediatric surgeons. Hospitals perform these procedures with a hospital admission or as an outpatient procedure.

The goal of this study was to analyze the contemporary distribution and preference of open and laparoscopic inguinal hernia repair in German pediatric surgical practice.

## 2. Materials and Methods

The study consisted of three parts: Part 1—A survey among all pediatric surgical departments; Part 2—The analysis of the national database on administrative claims data of all hospital admissions for Germany, provided by the Institute for the Remuneration System in Hospitals (InEK) [15]; Part 3—The analysis of the quality reports of the hospitals for Germany, published by the joined federal committee (G-BA) [16]. All data are obtained from the year 2019.

### 2.1. Survey among All Pediatric Surgical Departments of Germany

An anonymized online survey about the surgical concept of inguinal hernia repair (IHR) was sent to all department heads of the 89 pediatric surgical departments in Germany in 2019. The list of all pediatric surgical departments of Germany was obtained from the homepage of the German Society of Pediatric Surgery (DGKCH) [14]. The survey consisted of the following main questions:

- How many patients do you operate on with inguinal hernia per year?
- What technique is used for inguinal hernia repair?
- What kind of technique do you use for laparoscopic hernia repair?
- What is the reason to choose a specific technique?
- What kind of technique do you use for relapse?
- What size of instruments do you use?
- Who performs the surgery?
- Do you perform this surgery in incarcerated inguinal hernia?

The complete survey is available in Table 1.

### 2.2. Analysis of the National Database on Administrative Claims Data for Hospital Patients of Germany

Cumulative statistics on all hospital admissions were mined from the InEK (Institut für das Entgeldsystem im Krankenhaus, the German Institute for the Renumeration System of hospitals) [13]. These data contain all diagnoses and procedures of patients admitted to a German hospital, regarding age groups of the patients. Coding of the diagnoses is carried out with the International Classification of Diseases (ICD)-10 Code, German modification [17]. Procedures are coded using the German procedure classification (OPS) [18].

The data do not provide information on the specialty of the provider (e.g., pediatric surgeon, general surgeon, urologist) who performed the procedure.

This national database was analyzed for the year 2019. Data on all patients with the OPS 5-530 for IHR were extracted. Those patients with the code 5-530.02 for orchidofuniculysis and IHR were excluded.

### 2.3. Analysis of the Quality Reports of Germany

The yearly published quality reports contain the procedures performed for hospital-admitted patients and those treated on an outpatient basis for each hospital department. Some hospitals accumulate these data, e.g., on pediatric surgical patients together with general surgical patients. Only pediatric surgical datasets which were not accumulated with data from adult surgery were used in this study.

The quality reports of the hospitals of 2019 were analyzed regarding numbers of procedures of open and laparoscopic IHR performed in a pediatric surgical hospital department. For data protection rules, case frequencies below 4 were pooled and counted as one.

## 3. Results

### 3.1. Survey among All Pediatric Surgical Departments of Germany

Fifty-one of the 89 (58.4% recall) departments answered the questionnaire. Of these, 47.1% exclusively performed open IHR, 5.9% performed only laparoscopic IHR and both methods were performed in 47.1% of departments (Table 1).

Laparoscopic IHR was performed by fully trained specialists only in 51.6% of respondents, whereas it was considered a training procedure in 48.4% of the participating institutions. The main technique was percutaneous closure of the hernia under laparoscopic vision (33%). Intracorporal techniques using up to three trocars were employed in 63% of the queried departments. The choice between open and laparoscopic IHR was mainly determined by the age of the child (52%). Almost half of the departments used 3 mm instruments for the procedure, the others used 5 mm or 2 mm instruments. Of respondents, 20% used laparoscopic IHR in incarcerated hernia, 11% used it as the only technique for recurrent inguinal hernia (Table 1).

### 3.2. Analysis of the National Database on Administrative Claims Data for Hospital Patients of Germany

In 2019, 175,824 patients underwent inpatient IHR in Germany, 9718 (5.5%) of which were less than 18 years old. According to the national database, a total of 801 pediatric inguinal hernia repairs (under 18 years of age) were performed laparoscopically in the study interval.

**Figure 1:**
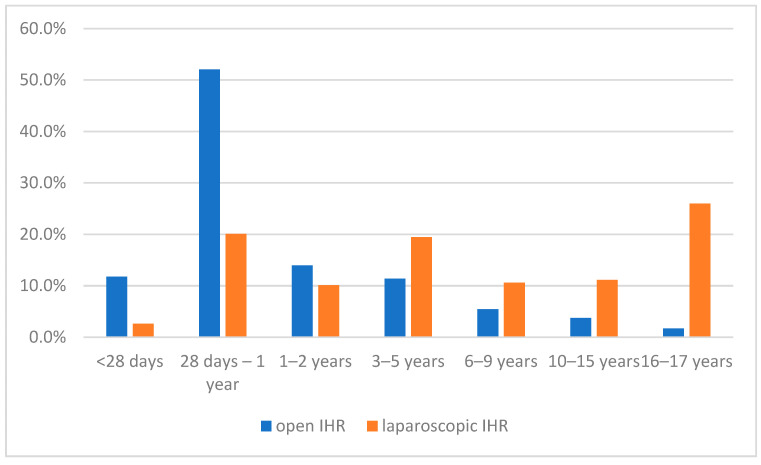
Distribution of age groups of children with open (*n* = 8926) and laparoscopic (*n* = 801) inguinal hernia repair.

Of the 9718 children with IHR, 18% were females, with a higher proportion of females (38%) in laparoscopic IHR. A total of 74% of all children with IHR were less than two years of age, compared to only 33% of the children with laparoscopic IHR (Table 2). The comparison of the age groups of the children with open and laparoscopic IHR is presented in Figure 1.

Of the patients with laparoscopic IHR, 90% had a main ICD code for inguinal hernia, meaning that they were admitted for IHR as the main reason for admission. Nearly 6% of them had a main ICD code for recurrent hernia, 6% had a code for incarcerated hernia and 24% had a code of bilateral inguinal hernia. Of the patients with open IHR, only 68% had a main code for inguinal hernia; the others had a hospital admission for other reasons. Of the patients admitted for open IHR, 3% had an ICD code for recurrent hernia, 12% for incarcerated hernia and 11% for bilateral hernia.

For the technique of laparoscopic IHR, the OPS codes differentiate between those with the use of any material and without. Using an OPS code with the use of any material requires a secondary code for the kind of material. Laparoscopic IHR with the use of any material was documented in 37% of the patients under the age of ten and 84% of the patients between ten and 17 years of age. Nonresorbable materials were documented in 33%, compared to (partial) resorbable materials in 23%. The code for the kind of used materials was not provided in 44% of all children. (Table 3)

### 3.3. Analysis of the Quality Reports of Germany

A total of 70 of the 89 (79%) pediatric surgical departments, four of the 42 small units (10%) and four of the 100 private practices (4%), who also have access to hospital beds, had public available data in their quality reports. University hospitals were represented in 23 of the 78 units (30%).

**Figure 2:**
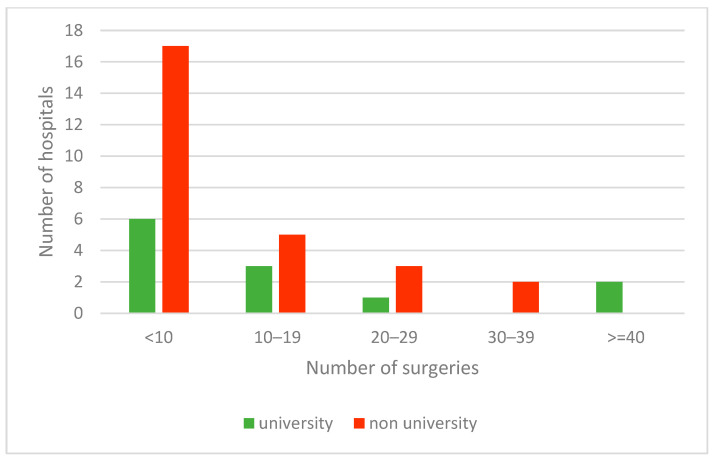
Number of procedures of laparoscopic inguinal hernia repair of university and non-university hospitals in 2019.

Forty of the 78 units (51%) and twelve of the 23 university hospitals (52%) performed laparoscopic IHR (Figure 2). The mean number for IHR in 2019 was 102 for all hospitals, 106 for university hospitals and 93 for non-university hospitals. The mean number for laparoscopic IHR was 12 for all hospitals, 19 for university hospitals and 9 for non-university hospitals. A total of 65% of all laparoscopic IHRs were performed by six hospitals, two of which were university hospitals, which performed 36% of those documented in the quality reports. The distribution of laparoscopic IHR regarding inpatient and outpatient procedures is provided in Table 4.

As in the InEK data, 48% of the codes used for laparoscopic IHR were codes with the use of any material; 52% were without. The corresponding codes for used material cannot be analyzed from these data.

## 4. Discussion

Our study gives an up-to-date account of the current practice of pediatric inguinal hernia in Germany. Consistent with previous studies [2], 82% of the children in our study were male and 86% were less than six years old.

We showed that open IHR with high ligation of the hernia sac is still the gold-standard technique in Germany. Laparoscopic IHR in children plays a minor role. However, there seems to be a trend towards more prevalent utilization of the laparoscopic technique. In a study of German administrative claims data for the years 2005–2017, laparoscopic IHR was used in boys for between 1% and 3% of cases and in girls for between 2% and 14% of cases for the different age groups [19]. While our analysis showed that the open technique is still used in more than 90% of all patients in 2019, the percentage of laparoscopic IHR increased to 6% of boys and 17% of girls. Nevertheless, these numbers are still lower than in a study from Japan with 26% of laparoscopic hernia repair in a cohort from 2010–2016 [20] and of a US study with data from 2009–2014 with a rate of 13% laparoscopic IHR [21].

Although a recent meta-analysis showed no overall superiority for laparoscopic IHR [22], some advantages are described. Laparoscopic IHR is seen advantageous for special conditions such as bilateral hernia [6,7] and incarcerated hernia [23,24]. In our series, the percentage of bilateral hernia in laparoscopic IHR was 24% and in open IHR it was 11%. For incarcerated inguinal hernia, laparoscopic IHR is regarded as easier to perform in literature. [23,24] In contrast to this, in our study only 6% of the laparoscopic IHR was for incarcerated hernia, whereas 12% of the open IHR was for incarcerated hernia.

Over the last decades, different methods for laparoscopic IHR have been described, including transperitoneal and extraperitoneal techniques. [1,25,26] In our survey 33% of pediatric surgeons prefer transperitoneal technique using only one trocar for the camera. Four different procedures with 2–3 trocars for intracorporal suturing with or without opening the peritoneum are performed in the study period in Germany. The use of mesh was not mentioned as a technique by pediatric surgeons. In contrast to this, the use of the code for IHR with any material is used by pediatric surgical departments, as seen in the quality reports. The analysis of the IHR in the InEK data leads to the conclusion that this is a coding problem, as in younger children no obligatory code for the kind of material is provided.

In the group of adolescents, 84% had surgery with the use of any material, and in 85% the additional obligatory OPS code for the material was given. As IHR in Germany is performed by pediatric and general surgeons, these numbers indicate that adolescents are operated on more often by general surgeons, since pediatric surgeons did not state this technique in our survey. This is in line with a survey among pediatric and general surgeons where technique was determined mostly by the preference of the surgeon, with general surgeons using mesh more often [27]. Regarding the medical impact of the mesh repair, a recent review of adolescent IHR concluded that there is no superiority of either method regarding recurrence rate. The mesh repair seemed to have a higher rate of chronic postoperative pain [28]. Further research regarding this topic should address children operated by general surgeons.

Laparoscopic IHR is not distributed equally in pediatric surgery in Germany, as 47% of the respondents of our survey and 49% of the pediatric surgical units with quality reports offered open surgery only. University medicine is generally seen as a promoter for innovation. However, in our study the percentage of university hospitals performing laparoscopic IHR was not higher than the percentage of non-university hospitals. Two university hospitals performed 36% of all laparoscopic procedures documented in the quality reports, indicating that these academic departments are protagonists for laparoscopic IHR.

Open IHR is an operation that is taught to the pediatric surgical trainee under direct supervision in the operation room. It is one of the first surgeries a trainee performs [29]. Laparoscopic IHR needs different training, but skills could be acquired within 12 months according to a Japanese study [30]. In our survey, laparoscopic IHR was performed in 48% of the hospitals by trainees.

Our study has several drawbacks. Most importantly, procedures of private practices are not entered into national or insurance databases, and quality reports do not distinguish between procedures for adults and underaged people. Therefore, our study focused on inguinal hernia repair performed in hospitals with pediatric surgery units.

We also have to address the above-mentioned coding problem. In our survey, pediatric surgeons did not use mesh, but in the data from InEK mesh repair was coded in 54.7% of the patients. When using a code for IHR with the use of any material, a secondary code has to be provided specifying the kind or material. This code was not given for 44.1% of the patients. In the group under the age of ten years, it was lacking in 82.9% of the patients. This is an indication of a coding problem, which is in line with the data of the quality reports, where 48.3% of the OPS codes performed by pediatric surgeons were for IHR with the use of any material, which is in contrast to the technical details provided in our survey.

## 5. Conclusions

Currently, only a minority of German children undergo inguinal hernia repair by laparoscopy. More training opportunities in the form of hands-on and video workshops may lead to more widespread employment of the laparoscopic technique.

## Figures and Tables

**Table 1:** Answers of the survey about laparoscopic inguinal hernia repair.

Question	Respondents	Answers	Percentage
What is your clinical setting?	52		
· Hospital department		49	94.2%
· Private practice with hospital beds		3	5.8%
Do you operate on children (0–14 years old) with indirect inguinal hernia in your department?	48		
· Yes		47	97.9%
· No		1	2.1%
How many patients with inguinal hernia do you operate on per year?	51		
· <50		6	11.8%
· <100		12	23.5%
· <150		17	33.3%
· More than 150		16	31.4%
What kind of technique do you use for inguinal hernia repair?	51		
· Open surgery only		24	47.1%
· Laparoscopic surgery only		3	5.9%
· Both techniques		24	47.1%
Which technique do you perform in laparoscopic inguinal hernia repair?	30		
· Percutaneous closure (1 trocar)		10	33.3%
· Intracorporal suture with cut of the peritoneum (2–3 trocar)		9	30.0%
· Intracorporal technique with sling (2–3 trocar)		2	6.7%
· Intracorporal suture without cut (2–3 trocar)		8	26.7%
· Other technique		4	13.3%
Indication for the kind of surgical technique of laparoscopic inguinal hernia repair.	31		
· Age of the child		16	51.6%
· Sex of the child		12	38.7%
· Pre-existing condition of the child		15	48.4%
· Preference of the surgeon		10	32.3%
· Request of parents		15	48.4%
· Other		8	25.8%
Do you perform laparoscopic inguinal hernia repair in incarcerated hernia?	30		
· Yes		6	20.0%
· No		24	80.0%
What kind of technique do you use in recurrent hernia?	46		
· Always open surgery		27	58.7%
· Always minimal-invasive surgery		5	10.9%
· Change of technique (dependent on the method used before)		13	28.3%
What kind of instruments do you use for minimal-invasive surgery?	30		
· 5 mm instruments		10	33.3%
· 3 mm instruments		14	46.7%
· 2 mm instruments		6	20.0%
What is your setting for minimal-invasive surgery?	26		
· Outpatient procedure		6	23.1%
· Inpatient procedure		13	50.0%
· Inpatient and outpatient procedure		7	26.9%
Who is performing the laparoscopic inguinal hernia repair?	31		
· Fully trained pediatric surgeons only		16	51.6%
· Fully trained pediatric surgeons and trainees		14	45.2%
· Trainees only		1	3.2%

**Table 2:** Age, gender and diagnoses of children with open and laparoscopic inguinal hernia repair.

	All		Open		Laparoscopic	
all	9718		8926	91.9%	801	8.2%
males	7968	82.0%	7363	84.0%	497	62.0%
females	1750	18.0%	1402	16.0%	304	38.0%
0–28 days	1067	11.0%	1046	11.7%	21	2.6%
29 days–1 year	4757	49.0%	4602	51.6%	161	20.1%
1–2 years	1343	13.8%	1263	14.1%	81	10.1%
3–5 years	1192	12.3%	1037	11.6%	156	19.5%
6–10 years	578	5.9%	493	5.5%	85	10.6%
11–15 years	424	4.4%	336	3.8%	89	11.1%
16–17 years	357	3.7%	149	1.7%	208	26.0%
Inguinal hernia as main diagnosis	7359	75.7%	6643	68.4%	718	89.6%
Recurrent inguinal hernia	225	2.3%	174	1.9%	47	5.9%
Incarcerated inguinal hernia	1089	11.2%	1028	11.5%	45	5.6%
Bilateral inguinal hernia	1175	12.1%	986	11.0%	190	23.7%

**Table 3:** Technique of laparoscopic inguinal hernia repair (IHR).

Age Group	All		0–9		10–17	
IHR without the use of any material	364	45.3%	317	62.9%	47	15.6%
with partial resection of hernia sac	68	8.5%	61	12.1%	7	2.3%
other	296	36.8%	256	50.8%	40	13.3%
IHR with the use of any material	440	54.7%	187	37.1%	253	84.3%
transperitoneal	354	44.0%	186	36.9%	168	56.0%
extraperitoneal	86	10.7%	1	0.2%	85	28.3%
Material						
not coded	194	44.1%	155	82.9%	39	15.4%
(partial) resorbable	103	23.4%	28	15.0%	75	29.6%
nonresorbable	143	32.5%	4	2.1%	139	54.9%

**Table 4:** Numbers of procedures of laparoscopic inguinal hernia repair (IHR) per pediatric surgical unit documented in the quality reports.

	Hospitals		Number of Laparoscopic IHR		
	Number	%	Median	1st Quartile	3rd Quartile
All hospitals	40		4	1	16.5
- inpatient	37	92.5%	3	1	15
- outpatient	13	32.5%	1	1	13
University hospitals	11		9.5	1.8	20
- inpatient	9	81.8%	8	2.5	19
- outpatient	5	45.5%	1	1	17
Non-university hospitals	29		3	1	13
- inpatient	28	96.6%	3	1	11.5
- outpatient	7	24.1%	1	1	1

## Data Availability

Data of the used quality reports of the hospitals of Germany can be found at https://www.g-ba-qualitaetsberichte.de/#/search, accessed 31 January 2022, data of the Remuneration System in Hospitals (InEK) can be retrieved at https://www.g-drg.de/, accessed 31 January 2022. All data of the survey are published in the article.
